# PRR34-AS1 promotes mitochondrial division and glycolytic reprogramming in hepatocellular carcinoma cells through upregulation of MIEF2: PRR34-AS1 promotes hepatocellular carcinoma growth

**DOI:** 10.3724/abbs.2024083

**Published:** 2024-05-22

**Authors:** Xuejing Yang, Huijing Feng, Jonghwa Kim, Gang Ti, Lin Wang, Kun Wang, Dong Song

**Affiliations:** 1 Cancer Center Shanxi Bethune Hospital Shanxi Academy of Medical Sciences Tongji Shanxi Hospital Third Hospital of Shanxi Medical University Taiyuan 030032 China; 2 Tongji Hospital Tongji Medical College Huazhong University of Science and Technology Wuhan 430030 China; 3 Department of Pharmaceutical Engineering Woosuk University Wanju Jeonbuk South Korea

**Keywords:** glucose metabolism reprogramming, hepatocellular carcinoma, lncRNA PRR34-AS1, mitochondrial division, proliferation

## Abstract

LncRNA PRR34-AS1 overexpression promotes the proliferation and invasion of hepatocellular carcinoma (HCC) cells, but whether it affects HCC energy metabolism remains unclear. Mitochondrial division and glycolytic reprogramming play important roles in tumor development. In this study, the differential expression of PRR34-AS1 is explored via TCGA analysis, and higher levels of PRR34-AS1 are detected in patients with liver cancer than in healthy individuals. A series of experiments, such as CCK-8, PCR, and immunofluorescence staining, reveal that the proliferation, invasion, glycolysis, and mitochondrial division of PRR34-AS1-overexpressing hepatoma cells are significantly promoted. TCGA analysis and immunohistochemistry reveal high expression of the mitochondrial dynamin MIEF2 in liver cancer tissues. Dual-luciferase reporter assays confirm that miR-498 targets and binds to mitochondrial elongation factor 2 (MIEF2). In addition, we show that PRR34-AS1 can sponge miR-498. Therefore, we further investigate the effects of the lncRNA PRR34-AS1/miR-498/MIEF2 axis on the growth, glucose metabolism, and mitochondrial division in hepatocellular carcinoma cells. A series of experiments are performed on hepatocellular carcinoma cells after different treatments. The results show that the proliferative activity, invasive ability, and glycolytic level of hepatocellular carcinoma cells are decreased in HCC cells with low PRR34-AS1 expression, and the miR-498 expression level is increased in these cells. Inhibition of miR-498 or overexpression of MIEF2 restored the proliferative activity, invasive ability, glycolysis, and mitochondrial division in hepatocellular carcinoma cells. Thus, PRR34-AS1 regulates MIEF2 by sponging miR-498, thereby promoting mitochondrial division, mediating glycolytic reprogramming and ultimately driving the growth and invasion of HCC cells. Furthermore, *in vivo* mouse experiments yield results similar to those of the *in vitro* experiments, verifying the above results.

## Introduction

Hepatocellular carcinoma (HCC) is one of the most common clinical malignant tumors, and its mortality rate ranks the third [1]. Liver cancer can be classified as a primary or secondary tumor, and 90% of primary liver cancers are liver hepatocellular carcinoma (LIHC) [2]. Surgical resection can be performed for early-stage liver cancer [3]. However, the proportion of patients with early-stage liver cancer is relatively small, and fewer than 15% of patients are good candidates for surgical treatment [4]. Treatment options for advanced HCC are severely limited. Systemic chemotherapy is the preferred treatment for advanced HCC. However, due to drug resistance mechanisms, treatment efficacy is low. Therefore, new targets are urgently needed to increase therapeutic efficacy and improve patient prognosis.

PRR34-AS1 is a newly discovered lncRNA. Previous studies have shown that it participates in various biological behaviors of liver cancer cells by regulating mRNA expression. PRR34-AS1 has been described as a proto-oncogene and is thus a new biological target for liver cancer research [ 5, 6]. Our previous study revealed that PRR34-AS1 can bind to miR-498 and that overexpression of PRR34-AS1 can upregulate the expressions of mitochondrial outer membrane 20 (TOMM20) and integrin subunit alpha 6 (ITGA6) and inhibit miR-498 expression. Thus, these factors may regulate the proliferation, migration, and invasion of hepatoma cells [6].

Mitochondrial division and fusion homeostasis are required for mitochondrial functions [7]. Previous studies in various malignant tumors have shown that mitochondrial division in tumor cells is abnormally activated. Activated mitochondrial division can further promote tumor progression [ 8 ‒ 10]. During the process of mitochondrial division, the dynamin protein Drp1 located in the cytoplasm is activated, translocates to the mitochondria and is located at the potential division site together with Fis1. Subsequently, the two cooperate to form a ring structure and promote the division of mitochondria [11]. Mitochondrial elongation factor 2 (MIEF2) is a key regulator of mitochondrial dynamics, and overexpression of MIEF2 induces the accumulation of Drp1 in the mitochondrial outer membrane [12], thus promoting mitochondrial division. Some studies have shown that mitochondrial division may play a role in tumors by generating energy products through the preferential use of glycolysis, increasing the biosynthesis of macromolecules, changing tumor metabolism, and promoting tumor cell proliferation [13].

The reprogramming of energy metabolism in tumor cells, especially glucose metabolism reprogramming, has received increasing attention from researchers [14]. In liver cancer, the reprogramming of glucose metabolism has continuously developed into one of the hallmarks of cancer [15], which is usually manifested by enhanced glycolysis and reduced oxidative phosphorylation. Glycolysis involves the metabolism of intracellular glucose into pyruvate through a series of enzyme-catalyzed reactions to produce ATP and NADH, both of which are very important for tumor cells; these molecules help meet the basic energy needs of cells to support survival and participate in tumor cell proliferation, transfer, antioxidant activity and other biological processes. In liver cancer, the enhancement of aerobic glycolytic activity is related to the upregulation of various glucose transporters and glycolytic enzymes [16].

Targeting liver cancer energy metabolism, especially glucose metabolism, may be a potentially effective treatment option. According to the research background described above, this study aimed to explore the mechanism by which the lncRNA PRR34-AS1 promotes the reprogramming of glucose metabolism by regulating mitochondrial division to drive the growth and invasion of liver cancer cells.

## Materials and Methods

### TCGA dataset

The RPKM values of the lncRNA PRR34-AS1 and MIEF2 in the TCGA database ( https://tcga-data.nci.nih.gov/tcga/) were downloaded. These data included 371 LICH tissues and 50 normal tissues. The expression levels of lncRNA PRR34-AS1 and MIEF2 were also analyzed in relation to the prognosis of patients with LIHC in the TCGA database.

### Cell culture and transfection

The SNU-182 cell line (CL-0610; Procell, Mannheim, Germany) was obtained in June 2022 and subjected to STR cell characterization. The SK-HEP-1 cell line (CL-0212; Procell) was obtained in January 2021 and subjected to STR cell characterization in February 2021. HCC cells were cultured at 37°C in DMEM (Four Seasons Biological Company, Hangzhou, China). The complete coding fragment of PRR34-AS1 was amplified by PCR, and the resulting fragment was ligated into the pcDNA3.1 vector (Invitrogen, Carlsbad, USA) to construct pcDNA3.1-PRR34-AS1 (a PRR34-AS1 overexpression vector). As a negative control, empty pcDNA3.1 was transfected (pc-NA). Short hairpin RNAs (shRNAs) targeting PRR34-AS1 (shRNA-PRR34-AS1, low expression of PRR34-AS1) and the corresponding negative control (sh-NC) were generated by GenePharma (Shanghai, China). The sequence of sh-NC was 5′-CCGGCACGATAAGACAATGTATTTCTCGAGAAATACATTGTCTTATCGTGTTTTTG-3ʹ and the sequence of sh-PRR34-AS1 was 5ʹ-CCGGCTCTAATAATGGAAAAAAATTTACTCGAGTAAATTTTTTTCCATTATTAGAGTTTTTG-3ʹ. Cell transfections were performed using Lipofectamine® 2000 transfection reagent (Invitrogen).

### Liver tissue specimens

Ten patients with HCC diagnosed at Shanxi Bethune Hospital between January 2018 and December 2021 were identified through the medical records of the inpatient and outpatient departments of Shanxi Bethune Hospital. Liver cancer tissue from the same patient was used as the experimental group, and normal adjacent tissue was used as the control group. Paraformaldehyde (4%) was used to fix the samples; sections were prepared and routinely dewaxed for immunohistochemical staining. The experiment protocols involving human subjects were approved by the Medical Ethics Committee of Shanxi Bethune Hospital (YXLL-2022-040).

### Mouse HCC transplantation tumor model

Male BALB/C-NU mice (6 weeks old) were purchased from Shanxi Medical University Laboratory Animal Center and housed in an SPF-grade environment. After 1 week of acclimatization, the mice were randomly divided into the shRNA-Control group, shRNA-PRR34-AS1 group, sh-PRR34-AS1+miR-498 antagomir group, and sh-PRR34-AS1+ov-MIEF2 group ( *n*=6). SK-HEP-1 cells were transfected with a PRR34-AS1 silencing virus (sh-PRR34-AS1; GenePharma) or a negative control (shRNA-Control; GenePharma). The transfected SK-HEP-1 cells were digested with trypsin and resuspended in PBS to a final concentration of 1×10 ^7^ cells/mL, and the cells (1 mL/only mice) were injected into the left side of the groin of the mice. Starting from the third week, the mice were injected intratumorally with 10 nmol/mouse miR-498 antagomir (GenePharma) or MIEF2-overexpressing adenovirus (1×10 ^10^ PFU/mouse, single injection; GenePharma). Then, the mice were sacrificed after 4 weeks, and the volume of the tumor tissue was measured. The study complied with the regulations of the Animal Control Committee of Shanxi Bethune Hospital affiliated to Shanxi Medical University (20230309), and every effort was made to minimize animal suffering and reduce the number of animals used.

### Immunohistochemistry (IHC)

The collected tissues were fixed in formalin for 24 h. The tissues were embedded in paraffin and sectioned. They were dewaxed in xylene prior to staining, hydrated in gradient alcohol, repaired by high-pressure citric acid, treated with hydrogen peroxide, and closed with BSA antigen. Afterward, the anti-MIEF2 antibody (1:100; Bioss, Beijing, China), anti-GLUT1 antibody (1:200; Abcam, Cambridge, UK), anti-HK2 antibody (1:200; Proteintech, Wuhan, China), anti-PKM2 antibody (1:200; Proteintech), and anti-LDHA antibody (1:200; Servicebio, Wuhan, China) were added and incubated overnight. The HRP-labeled goat anti-rabbit secondary antibody (1:100; Servicebio) was then added and reacted at 28°C. Freshly prepared DAB was used for color development, and hematoxylin was used for restaining. The sections were dehydrated in gradient alcohol and xylene, sealed, dried and then analyzed under a microscope. The expression levels of MIEF2, GLUT1, HK2, PKM2, and LDHA in the tissues were assessed according to the percentage of cells stained.

### Transmission electron microscopy (TEM)

The tumor tissue was prefixed with 3% glutaraldehyde for 24 h and 1% osmium tetroxide for another 2 h, dehydrated with acetone, permeabilized, embedded, prepared in ultrathin sections of approximately 60‒90 nm, and collected onto a Formvar copper grid 200 mesh. The copper mesh was first stained with uranium acetate in the dark for 10‒15 min and then with lead citrate without CO _2_ for 1‒10 min. The copper mesh sections were placed in a copper mesh box and dried at room temperature overnight. Images were acquired and analyzed with a JEM-1400FLASH transmission electron microscope (JEOL, Tokyo, Japan).

### CCK-8 assay

After logarithmic growth, diluted and digested cells and a cell suspension were prepared in complete culture medium. A total of 100 μL/well was inoculated into a 96-well plate and incubated at 37°C with 5% CO _2_. After the cells were cultured for 24 h, the medium was replaced by fresh medium, and 10 μL of CCK-8 reagent (Biosharp, Guangzhou, China) was added to each well. The plates were incubated in the dark, and cell viability was calculated based on the absorbance at 450 nm detected via a microplate reader (spectra max PLUS 384; Molecular Devices, Shanghai, China).

### Wound healing assay

Treated HCC cells in the logarithmic growth phase were digested and resuspended. The number of cells was counted, and 5×10 ^5^ cells were seeded in each well of a 6-well plate. After 24 h of incubation, a scratch was made across the cell monolayer of each plate, and the plate was washed with PBS to remove the exfoliated cells. Serum-free medium was added, and the cells were photographed. The cells were incubated in an incubator for 24 h. The relative cell migration distance was calculated as follows: relative cell migration distance (μm)=24 h migration distance–0 h migration distance.

### Transwell invasion assay

Pre-chilled at 4°C with 1:3 dilution of Matrigel (CORNING, Suzhou, China) was added to the Transwell upper chamber, spread well, and dried at 37°C for 70 min. Groups were prepared with cell suspensions, and the cell concentration was adjusted to 4×10 ^4^ cells/mL, 200 μL of cell suspension was added to the upper chamber, and 500 μL of medium containing 20% FBS was added to the lower chamber as a chemotactic factor. The small chambers were incubated at 5% CO _2_ and 37°C for 24 h. The cells in the upper chamber were wiped off with cotton swabs, rinsed with PBS, fixed with methanol, and stained with 0.1% crystal violet (BOMEI, Hefei, China). Three fields of view of each well were selected and photographed under a light microscope (Olympus, Tokyo, Japan), and the number of migrated cells in each group was counted.

### Glucose uptake assay

The culture medium was collected before and after 24 h of culture. Glucose content in the culture medium was measured using a glucose assay kit (Nanjing Jiancheng Biological Company, Nanjing, China) according to the manufacturer’s instructions. The decrease in the amount of glucose in the culture medium reflects the amount of glucose taken up by the cells.

### Lactate production assay

Each group of cells was inoculated in a 6-well plate and incubated. The old medium was discarded, and new medium was added. The medium was collected from each sample before the assay, and the absorbance was measured using a multifunctional enzyme marker and a lactate assay kit (Nanjing Jiancheng Biological Company) to analyze the lactate concentration based on the protein concentration.

### ATP content assay

The ATP levels in the cells were measured with an Enhanced ATP Assay kit (Beyotime, Shanghai, China). After the medium was discarded and the cells were washed with PBS, the cells were inoculated in plates, and lysis buffer was added to each well. After sufficient lysis, the sample was centrifuged, and the supernatant was removed for subsequent assays. ATP working solution and sample were added to a 96-well white microtiter plate. Chemiluminescence values were measured for each well, and ATP concentrations were calculated.

### Mitochondrial division of hepatoma cells

After different treatments, HCC cells were seeded in unique dishes for laser confocal microscopy and cultured overnight. After the cell culture medium was discarded, the cells were washed with PBS, prewarmed mitochondrial fluorescence staining solution (Mio-Tracker Red) was added, and incubated (37°C for 30 min). After the staining solution was discarded, the sections were washed with PBS. Finally, the cells were photographed under a laser confocal microscope (VS200; Olympus) for subsequent analysis.

### Dual-luciferase reporter assay

The complete 3′UTR sequence of *MIEF2* containing the miR-498 binding site was amplified, subcloned and inserted into the pSI-Check2 vector to generate a wild-type (wt) vector for MIEF2 (h-MIEF2-3′UTR-wt). The 3′UTR sequence of *MIEF2*, which was designed not to contain the miR-498 binding site, was amplified, subcloned and inserted into the pSI-Check2 vector to generate a mutant vector for MIEF2 (h-MIEF2-3′UTR-mu). The cells and the target plasmid h-MIEF2-3′UTR were prepared for transfection. DMEM/h-MIEF2-3′UTR and hsa-miR-498-5p/negative control (NC) were thoroughly mixed. DMEM and transfection reagent (Hanheng Biotechnology, Shanghai, China) were fully mixed. The two solutions were mixed thoroughly. Before transfection, the medium was replaced by fresh medium, and the transfection mixture was added. After culture, the medium was substituted with fresh medium, and the cells were collected for detection after 48 h of transfection. A Dual-luciferase Assay System kit (Promega, Madison, USA) was used to measure and record the *Renilla* luciferase activity value, which is the reporter gene luminescence value.

### RT-PCR

Total RNA from SK-HEP-1 cells, SNU-182 cells, and tumor tissue was extracted using Trizol reagent (Invitrogen). Reverse transcription was performed to synthesize cDNA. cDNA, SYBR Green PCR Master Mix (Applied Biosystems, Foster City, USA) and gene-specific primers were used for RT-PCR. The forward primer for miR-498 was 5′-TATATTTCAAGCCAGGGGGCGTTT-3′, and the reverse primer was obtained from Sangon Biotech (Shanghai, China). Other primer sequences are shown in Table 1. All primers were synthesized by Sangon Biotech. The reaction conditions were as follows: 95°C for 30 s, 95°C for 10 s, and 72°C for 15 s for 40 cycles. The relative expression of each target gene was calculated via the 2 ^–ΔΔCT^ method.

**Table:** **Table 1** Sequences of primers used in this study

Gene	Primer sequence (5′→3′)
*PRR34-AS1*	Forward: GTTCCAGTGTCATCGTGTGG
Reverse: AGATTGCATTGGCTTGCTTT
*GLUT1*	Forward: GGCCAAGAGTGTGCTAAAGAA
Reverse: ACAGCGTTGATGCCAGACAG
*HK2*	Forward: GAGCCACCACTCACCCTACT
Reverse: GAGCCACCACTCACCCTACT
*PKM2*	Forward: ATTATTTGAGGAACTCCGCCGCCT
Reverse: ATTCCGGGTCACAGCAATGATGG
*LDHA*	Forward: TTGACCTACGTGGCTTGGAAG
Reverse: GGTAACGGAATCGGGCTGAAT
*MIEF2*	Forward: AGCACCGAAAGCAAGGAGAAGAAG
Reverse: CACCACCACCGAGAGCATCAAG
*β-actin*	Forward: TGTATGCCTCTGGTCGTACC
Reverse: CAGGTCCAGACGCAGGATG

### Western blot analysis

RIPA cell lysis buffer was added to lyse the cells and tumor tissue, which were subsequently centrifuged to collect the protein supernatant. SDS loading buffer was added, and the samples were boiled in boiling water. SDS-PAGE was used to separate proteins, which were transferred to PVDF membranes. Membranes were then blocked with 5% skim milk powder at room temperature. After incubation with primary antibodies, including anti-Fis1 (10956-1-AP, 1:2000; Wuhan Sanying Biological Co., Wuhan, China), anti-Drp1 (12957-1-AP, 1:2000; Wuhan Sanying Biological Co.), anti-MIEF2 (BS-12633R, 1:100; Beijing Boaosen Biotechnology Co., Beijing, China), and anti-β-actin (81115-1-RR, 1:100,000; Wuhan Sanying Biological Co.), the membranes were washed with PBS three times, and incubated with goat anti-rabbit IgG (H+L) secondary antibody (1:5000; ABclonal, Wuhan, China) for 1 h at room temperature, developed by enhanced chemiluminescence (ECL; Zenbio, Shanghai, China), and the bands were exposed with Fluorescence Image Analysis System Software V2.0 (Tanon, Shanghai, China), and the results were scanned by Gel-Pro analyzer4 software and expressed as the integrated optical density (IOD) of the target protein.

### Statistical analysis

Data were analyzed with GraphPad Prism 8 (GraphPad Software, San Diego, USA) and are presented as the mean±SD. Comparisons between two groups were performed using Student’s t tests or one-way ANOVA. Differences with *P* values less than 0.05 were considered to indicate statistical significance.

## Results

### Effects of PRR34-AS1 expression on the proliferation and invasion of hepatocellular carcinoma cells

**Figure 1:**
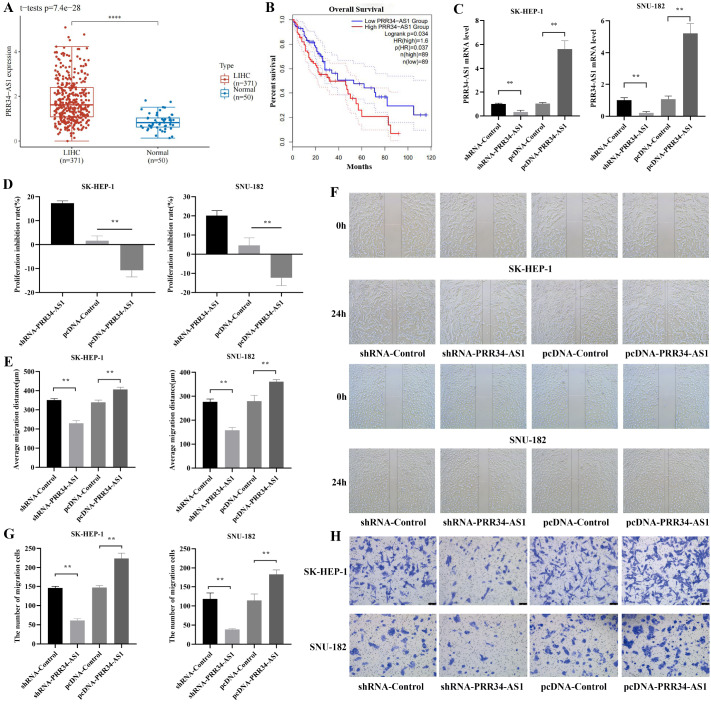
Figure 1 Effect of the PRR34-AS1 on the proliferation, migration, and invasion of HCC cells (A) The expression of PRR34-AS1 in patients with HCC. (B) Correlation between PRR34-AS1 expression and survival in patients with HCC. (C) The expression of PRR34-AS1 in hepatoma cells. (D) Proliferative activity of the LIHC cells in each group. (E) The average migration distance of the LIHC cells in each group at 24 h. (F) Microscopic observation of the migration of the LIHC cells in each group (40×). (G) The number of LIHC cells that passed through the Transwell in each group. (H) Results of the Transwell invasion assay of LIHC cells in each group (crystal violet staining). Scale bar: 100 μm. **P<0.01, ****P <0.0001.

The expression of PRR34-AS1 was significantly greater in HCC tissues than in normal tissues ( *P*<0.01; Figure 1A), and there was a difference in survival time between patients with high PRR34-AS1 expression and those with low PRR34-AS1 expression *(P* <0.05; Figure 1B). A series of transfections were performed on liver cancer cells to silence or overexpress PRR34-AS1. Compared with that in the control group, the expression of PRR34-AS1 in the shRNA-PRR34-AS1 and pcDNA-PRR34-AS1 groups was clearly decreased and increased, respectively ( *P*<0.01; Figure 1C). The proliferation, migration, and invasion abilities of hepatoma cells were assessed via CCK-8, wound healing, and transwell invasion assays, respectively. The proliferation of the cells in the pcDNA-PRR34-AS1 group was greater than that in the pcDNA-Control group ( *P*<0.01; Figure 1D). The mean migration distance of cells in the shRNA-PRR34-AS1 group was significantly shorter than that in the shRNA-Control group, and the migration distance of cells in the pcDNA-PRR34-AS1 group was significantly greater than that in the pcDNA-Control group ( *P*<0.01; Figure 1E,F). The number of cells that passed through the Transwell membrane in the pcDNA-PRR34-AS1 group was greater than that in the pcDNA-Control group. There were significantly fewer cells in the shRNA-PRR34-AS1 group than in the control group ( *P*<0.01; Figure 1G,H).

### Effect of PRR34-AS1 expression on glycolytic reprogramming in hepatocellular carcinoma cells

**Figure 2:**
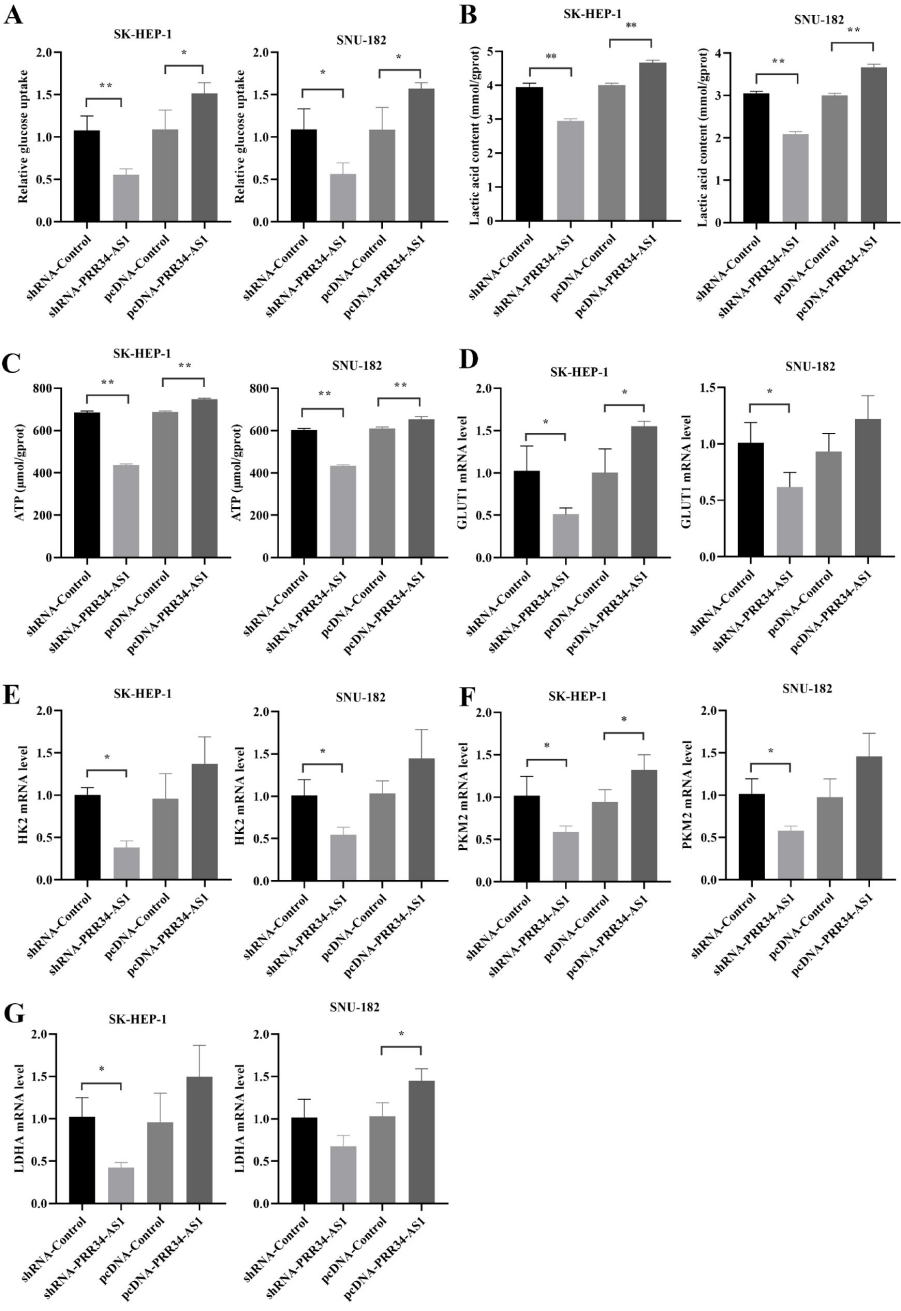
Figure 2 Effect of PRR34-AS1 expression on glycolytic reprogramming in HCC cells (A) Statistics of relative glucose intake in LIHC cells. (B) Lactate content in LIHC cells. (C) ATP content in LIHC cells. (D) GLUT1 mRNA expression in LIHC cells. (E) mRNA expression of HK2 in LIHC cells. (F) mRNA expression of PKM2 in LIHC cells. (G) LDHA mRNA expression in LIHC cells. *P<0.05, **P<0.01.

The effect of the PRR34-AS1 on cellular glucose metabolism reprogramming was evaluated by detecting glucose uptake and lactate and ATP levels in cells. Hepatoma cells in the pcDNA-PRR34-AS1 group had the highest glucose uptake, lactate content, and ATP content, which were markedly greater than those in the pcDNA-Control group ( *P*<0.05). Conversely, HCC cells in the shRNA-PRR34-AS1 group had the lowest glucose uptake, lactate content, and ATP content, and these values were significantly lower than those in the shRNA-Control group ( *P*<0.05; Figure 2A‒C). Glycolytic pathway-related kinase proteins were detected using PCR assays. Among the four groups of cells, the mRNA expression levels of GLUT1, HK2, PKM2, and LDHA were the highest in both SK-HEP-1 cells and SNU-182 cells. In contrast, the expression levels of these proteins were the lowest in the shRNA-PRR34-AS1 group ( Figure 2D‒G).

### Effects of PRR34-AS1 expression on mitochondrial division in liver cancer cells

**Figure 3:**
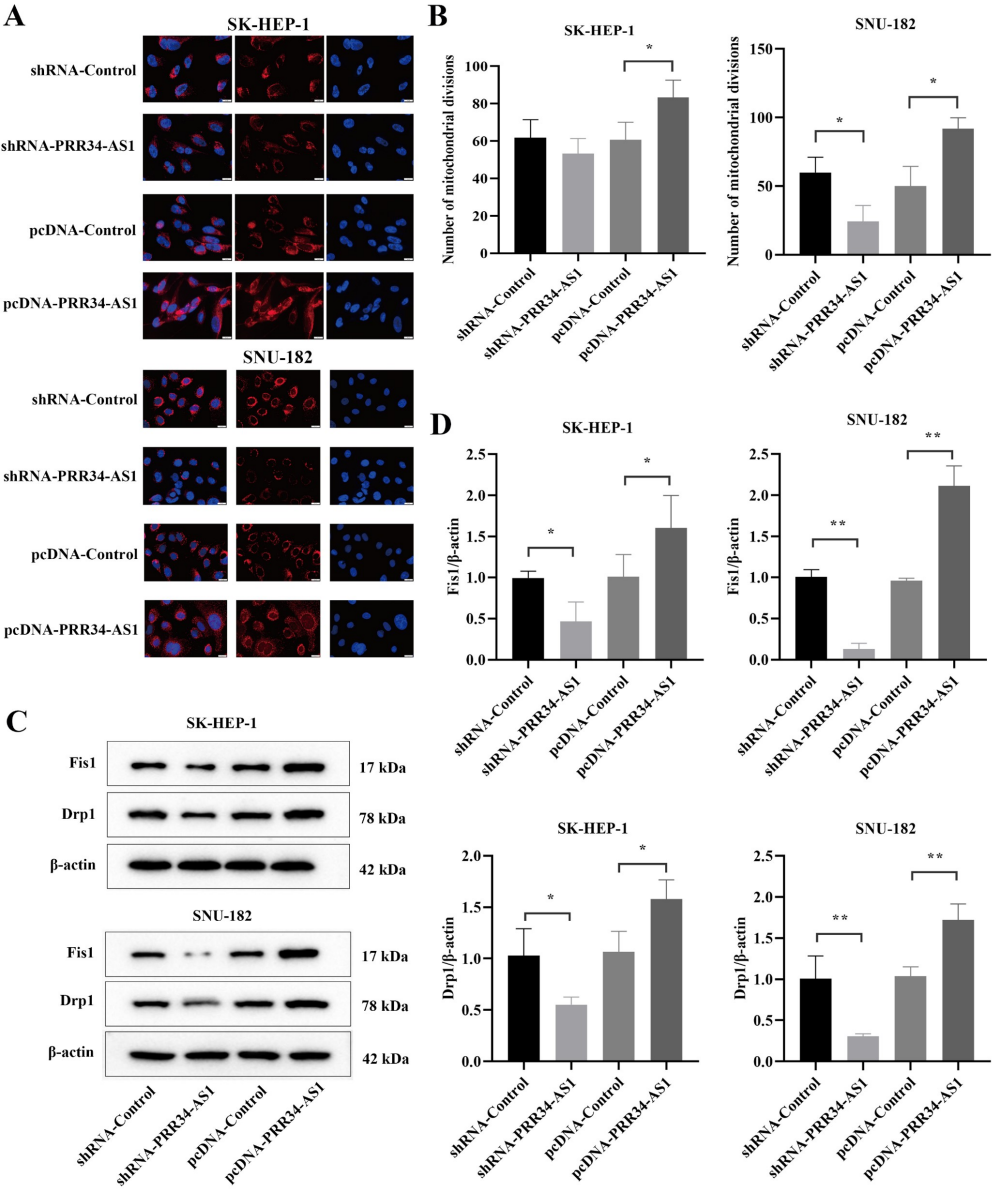
Figure 3 Effect of PRR34-AS1 expression on mitochondrial division (A) Immunofluorescence staining was used to detect the mitochondrial division of the hepatocellular carcinoma cells in each group (scale bar: 20 μm). (B) Statistics of the number of mitochondrial divisions in each group. (C) The expressions of Fis1 and Drp1 were detected by western blot analysis (the strips were cut for visualization). (D) Statistical analysis of Fis1 and Drp1 protein expressions in liver cancer cells from each group. *P<0.05, **P <0.01.

The enhanced glycolysis in tumor cells may be a result of the inhibition of oxidative phosphorylation following irreversible damage to mitochondrial function, and the dynamic balance of mitochondrial division and fusion is an important prerequisite to ensure the physiological function of mitochondria. The fluorescent probe MitoTracker Red was used to specifically label mitochondria to observe the mitochondrial division of the LIHC cells in each group. Immunofluorescence staining revealed that mitochondrial division occurred more readily in the pcDNA-PRR34-AS1 group of HCC cells than in the pcDNA-Control group, and mitochondrial division was suppressed in the shRNA-PRR34-AS1 group ( Figure 3A). In SK-HEP-1 cells, the proportion of mitochondrial division in the pcDNA-PRR34-AS1 group was the highest. In SNU-182 cells, the proportion of cells undergoing mitochondrial division in the pcDNA-PRR34-AS1 group was the greatest ( Figure 3B). In LIHC cells, the expression levels of Drp1 and Fis1 in the pcDNA-PRR34-AS1 group were the highest and were significantly greater than those in the pcDNA-Control group ( *P*<0.05). The Fis1 and Drp1 protein expression levels were the lowest in the shRNA-PRR34-AS1 group, and the levels were significantly lower than those in the shRNA-Control group ( *P*<0.05; Figure 3C,D).

### *MIEF2* is a target gene of miR-498 and is regulated by the PRR34-AS1

**Figure 4:**
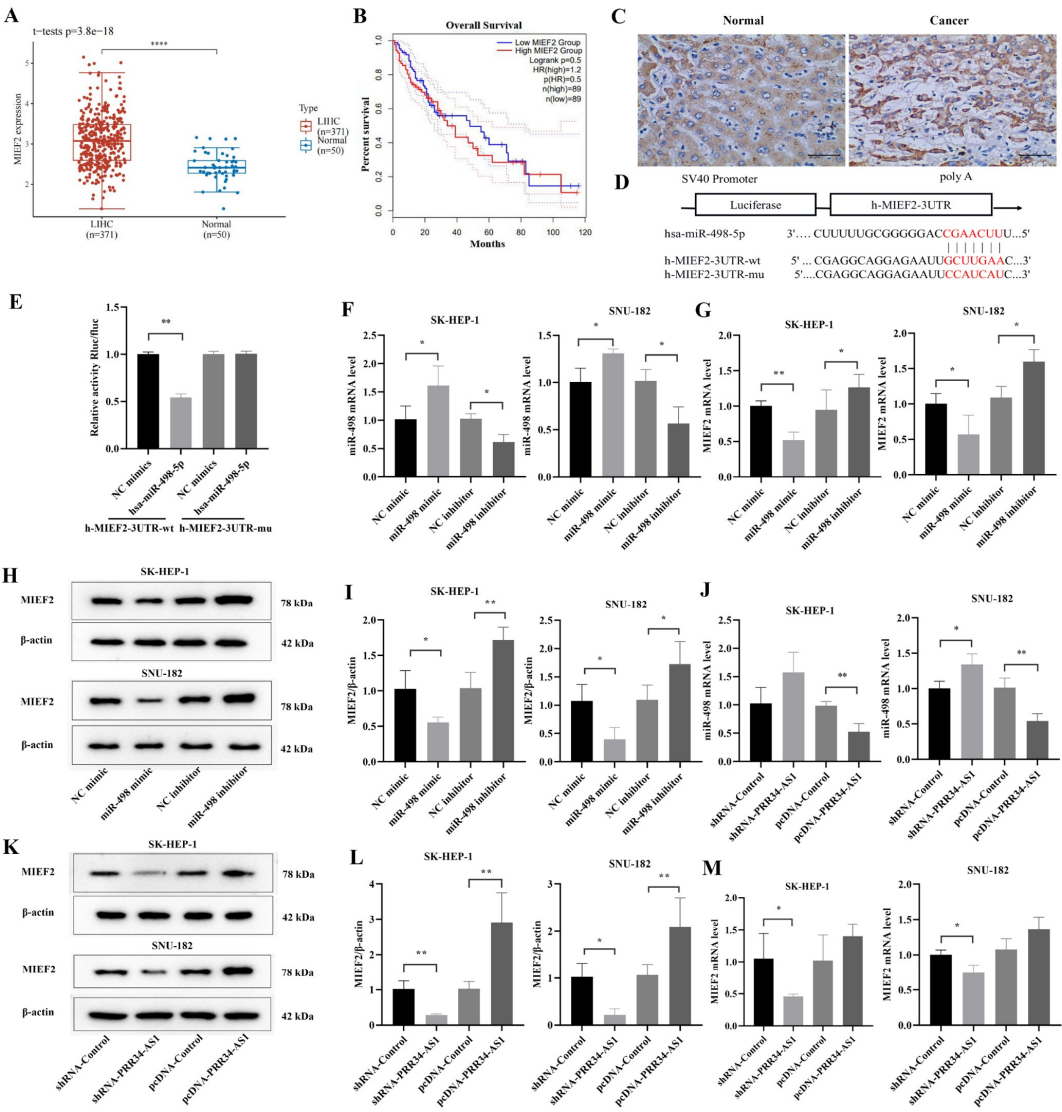
Figure 4 PRR34-AS1/miR-498/MIEF2 axis targeting relationship analysis (A) Differential expression of MIEF2 in patients with HCC. (B) Correlation between MIEF2 expression and survival in patients with HCC. (C) Differential expression of MIEF2 in HCC tissues and adjacent tissues (scale bar: 50 μm). (D) Schematic diagram of miR-498-5p binding to h-MIEF2-3′UTR target sites. (E) Dual luciferase reporter assay for the interaction between miR-498-5p and the h-MIEF2-3′UTR. (F) The expression of miR-498 in LIHC cells. (G) The mRNA expression of MIEF2 in LIHC cells. (H,I) MIEF2 expression was detected by western blot analysis (the strips were cut for visualization). (J) Expression level of miR-498 in LIHC cells. (K,L) MIEF2 protein expression in cells was tested via western blot analysis (the strips were cut for visualization). (L,M) MIEF2 mRNA expression in LIHC cells. *P<0.05, ** P<0.01.

MIEF2 is a mitochondrial outer membrane protein with a regulatory role in mitochondrial fission. The differential expression and prognostic value of MIEF2 in patients with HCC were analyzed via the TCGA database. MIEF2 was expressed at significantly greater levels in liver cancer tissues than in normal tissues ( Figure 4A), and the overall survival rate was lower in patients with high MIEF2 expression than in those with low MIEF2 expression ( Figure 4B). Immunohistochemistry verified that the expression of MIEF2 was significantly greater in HCC tissues than in normal tissues ( *P*<0.01). The percentage of positive MIEF2 expression among all adjacent tissues was 10%, but that for liver cancer tissue was 80% ( Figure 4C). Previous studies demonstrated that the lncRNA PRR34-AS1 sponges miR-498. Through bioinformatics online software prediction, it was found that MIEF2 binds to the target site of miR-498 ( Figure 4D). Compared with NC mimics, hsa-miR-498-5p mimics significantly decreased luciferase activity in the h-MIEF2-3UTR-WT group ( *P*<0.05; Figure 4E). There was a binding effect between the two, indicating that they were interconnected. Therefore, we surmised that the effect of the lncRNA PRR34-AS1 on mitochondrial division and glucose metabolism in HCC cells is achieved by sponging miR-498 and further regulating the expression of MIEF2.

The mRNA expression level of miR-498 in hepatoma cells in the miR-498 mimic group was significantly increased ( *P*<0.05), while that in the miR-498 inhibitor group was significantly decreased ( *P*<0.05). Moreover, the mRNA expression level of MIEF2 in the miR-498 mimic group was markedly lower than that in the NC mimic group ( *P*<0.05). The mRNA expression level of MIEF2 in the miR-498 inhibitor group was notably lower, and the expression level of miR-498 was markedly greater than that in the NC inhibitor group ( *P*<0.05; Figure 4F,G). The results of the western blot analysis of the protein expression of MIEF2 were consistent with the PCR results ( Figure 4H,I). To verify the regulatory effect of the lncRNA PRR34-AS1/miR-498/MIEF2 axis, the expression levels of miR-498 and MIEF2 in different groups of LIHC cells were measured by PCR and western blot analysis ( Figure 4J‒M). The expression level of miR-498 in LIHC cells in the pcDNA-PRR34-AS1 group was notably lower than that in the pcDNA-Control group ( *P*<0.05). In contrast, a noticeable increase in MIEF2 protein expression was observed in LIHC cells in the pcDNA-PRR34-AS1 group ( *P*<0.05). MiR-498 expression level was greater in the shRNA-PRR34-AS1 group than in the control group, while MIEF2 expression level was lower in the shRNA-PRR34-AS1 group than in the control group.

### Effect of the PRR34-AS1/miR-498/MIEF2 axis on the proliferation, invasion and glycolytic reprogramming of hepatocellular carcinoma cells

**Figure 5:**
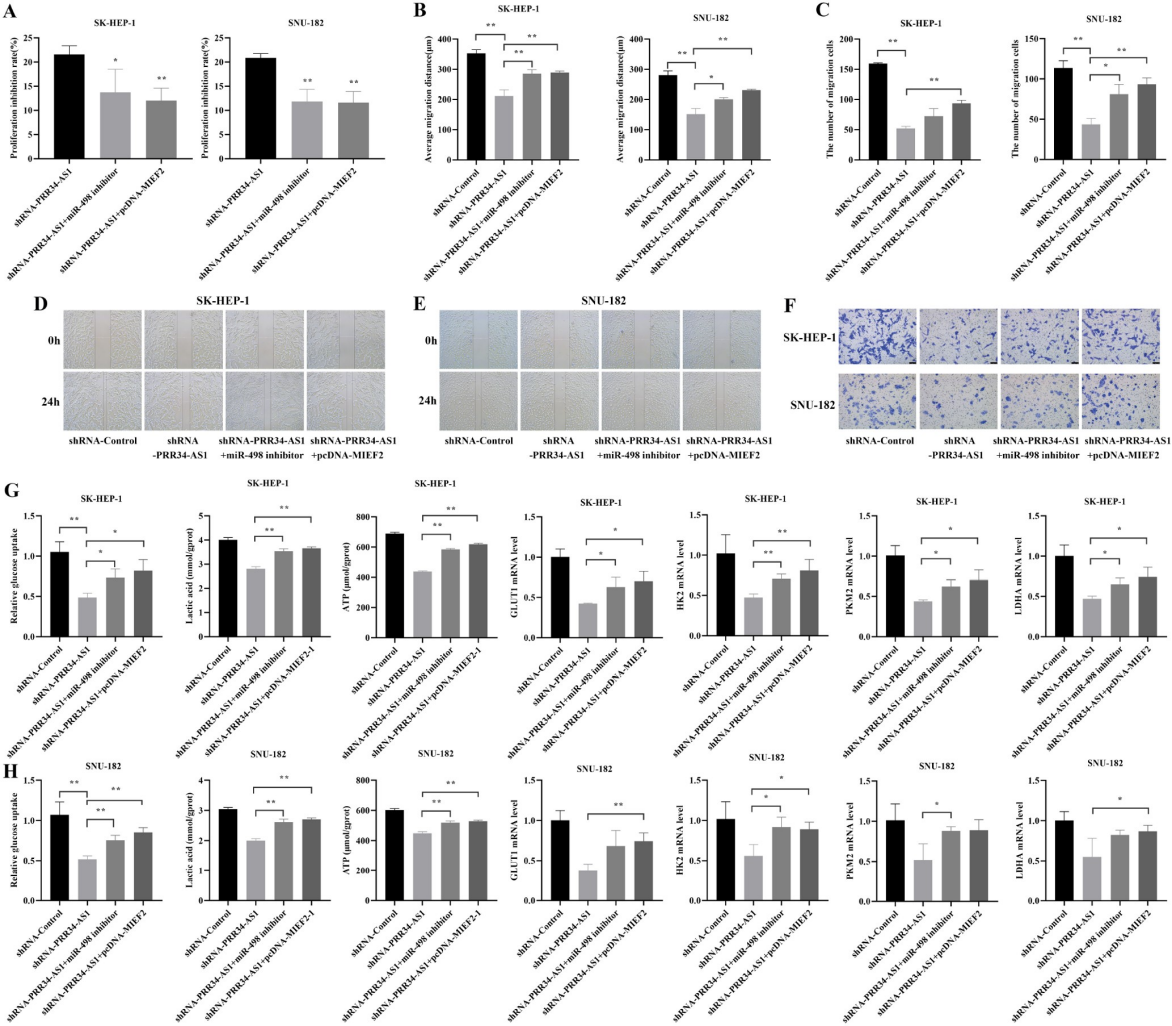
Figure 5 Effect of the PRR34-AS1/miR-498/MIEF2 axis on the proliferation, invasion and glycolytic reprogramming of HCC cells (A) The proliferative activity of the LIHC cells in each group. (B) The mean migration distance of the LIHC cells in each group at 24 h. (C) The number of LIHC cells that passed through the Transwell membrane in each group. (D,E) The migration of LIHC cells was observed under a microscope (100×). (F) Results of the Transwell invasion test of LIHC cells (crystal violet staining) (40×). (G) Relative glucose uptake, lactate content, ATP content and GLUT1, HK2, PKM2 and LDHA mRNA expressions in SK-HEP-1 cells. (H) Relative glucose uptake, lactate content, ATP content and GLUT1 , HK2, PKM2 and LDHA mRNA expressions in SNU-182 cells. *P<0.05, **P <0.01.

Compared with those in the shRNA-PRR34-AS1 group, the proliferation inhibition rates of SK-HEP-1 and SNU-182 cells in the shRNA-PRR34-AS1+miR-498 inhibitor group were notably decreased ( *P*<0.05). Inhibition of miR-498 expression or MIEF2 overexpression can upregulate the inhibitory effect on proliferation caused by PRR34-AS1 downregulation ( Figure 5A). Compared to those in the shRNA-Control group, the migration distances of LIHC cells in the shRNA-PRR34-ASI group were significantly decreased. However, the migration distance of LIHC cells in the shRNA-PRR34-AS1+miR-498 inhibitor group was notably greater than that in the control group ( *P*<0.05). The same effect was also observed in the shRNA-PRR34-AS1+pcDNA-MIEF2 group ( Figure 5B,D,E). The numbers of cells in the shRNA-PRR34-AS1+miR-498 inhibitor group and shRNA-PRR34-AS1+pcDNA-MIEF2 group that crossed the Transwell chamber were notably greater than those in the shRNA-Control group ( *P* <0.01; Figure 5C,F).

Compared with those in the shRNA-PRR34-AS1 group, both the shRNA-PRR34-AS1+miR-498 inhibitor group and the shRNA-PRR34-AS1+pcDNA-MIEF2 group exhibited significantly increased glucose intake and lactate and ATP content in the cells ( *P*<0.01; Figure 5G,H). PCR was used to measure the expression levels of glycolytic pathway-related kinases ( Figure 5G,H). The expression levels of these proteins in LIHC cells in the shRNA-PRR34-AS1 group were lower than those in the control group. These proteins were upregulated in the shRNA-PRR34-AS1+miR-498 inhibitor group and shRNA-PRR34-AS1+pcDNA-MIEF2 group compared to the shRNA-PRR34-AS1 group.

### Effect of the PRR34-AS1/miR-498/MIEF2 axis on mitochondrial kinetics in hepatocellular carcinoma cells

**Figure 6:**
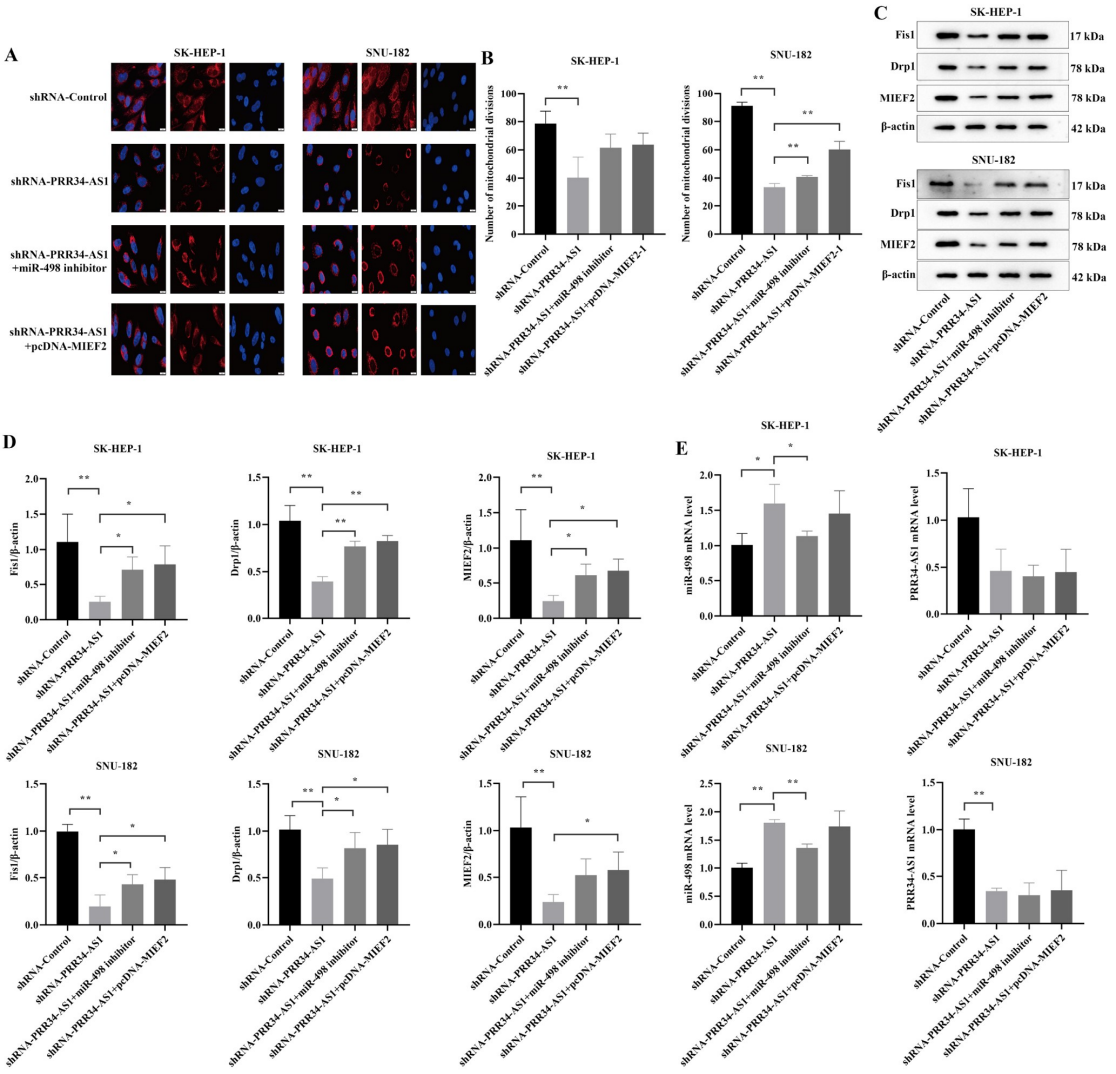
Figure 6 Effect of the PRR34-AS1/miR-498/MIEF2 axis on mitochondrial division in liver cancer cells (A) Mitochondrial division in LIHC cells in each group (scale bar: 20 μm). (B) Statistics of the number of mitochondrial divisions in each group. (C) Expressions of mitochondrial division-related proteins were measured by western blot analysis (the strips were cut for visualization). (D) Fis1, Drp1, and MIEF2 protein expressions in LIHC cells. (E) miR-498 and PRR34-AS1 mRNA expressions in LIHC cells. *P<0.05, **P<0.01.

shRNA-PRR34-AS1 reduced mitochondrial disruption in LIHC cells compared with that in shRNA-Control cells ( *P*<0.01). In the shRNA-PRR34-AS1+miR-498 inhibitor group and shRNA-PRR34-AS1+pcDNA-MIEF2 group, the number of mitochondrial divisions in LIHC cells was increased. The degree of mitochondrial division in the shRNA-PRR34-AS1 group was significantly lower than that in the other three groups, as determined via immunofluorescence staining ( Figure 6A,B). The protein expression levels of Fis1, Drp1, and MIEF2 in the shRNA-PRR34-AS1 group were the lowest and were notably lower than those in the shRNA-Control group ( *P*<0.01). Compared to those in the shRNA-PRR34-AS1 group, the expression levels of these proteins were greater in the shRNA-PRR34-AS1+miR-498 inhibitor group and shRNA-PRR34-AS1+pcDNA-MIEF2 group ( Figure 6 C,D).

The expression levels of PRR34-AS1 in LIHC cells in the shRNA-PRR34-AS1, shRNA-PRR34-AS1+miR-498 inhibitor, and shRNA-PRR34-AS1+pcDNA-MIEF2 groups were lower than those in the shRNA-Control group. In all three groups, PRR34-AS1 was expressed at similar levels ( Figure 6E). In addition, the expression of miR-498 in the shRNA-PRR34-AS1 group was markedly greater than that in the shRNA-Control group and shRNA-PRR34-AS1+miR-498 inhibitor group ( *P*<0.05). However, its expression in the shRNA-PRR34-AS1 group did not differ significantly from that in the shRNA-PRR34-AS1+pcDNA-MIEF2 group, indicating that high expression of MIEF2 could not reverse the increase in the expression of miR-498 mediated by PRR34-AS1. In conclusion, the lncRNA PRR34-AS1 affects the mitochondrial kinetics of LIHC cells through the targeted regulation of MIEF2 by miR-498.

### Effect of the PRR34-AS1/miR-498/MIEF2 axis on mitochondrial kinetics and glycolytic reprogramming in hepatocellular carcinoma mice

**Figure 7:**
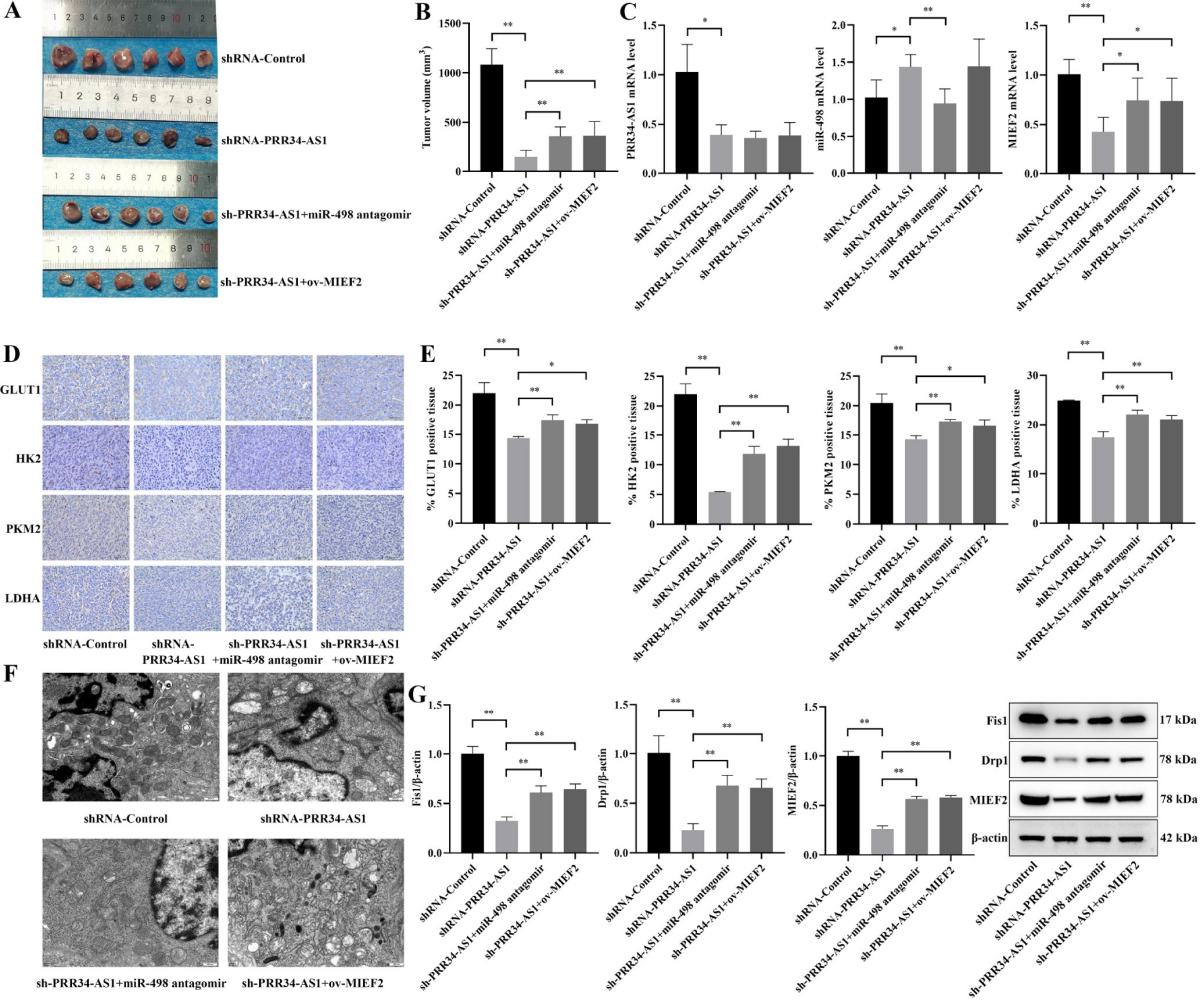
Figure 7 Effect of the PRR34-AS1/miR-498/MIEF2 axis on mitochondrial kinetics and glycolytic reprogramming in hepatocellular carcinoma mice (A) Tumor size. (B) Tumor volume. (C) The expressions of PRR34-AS1, miR-498, and MIEF2 in tumor tissue. (DE) Differential expressions of GLUT1, HK2, PKM2, and LDHA in tumor tissue (scale bar: 40 μm). (F) Transmission electron microscopy of tumor tissue (20,000×; scale bar: 500 nm). (G) Expressions of mitochondrial division-related proteins were measured via western blot analysis (the strips were cut for visualization), and the results of the statistical analysis of Fis1, Drp1, and MIEF2 protein expressions are shown. *P<0.05, **P <0.01.

Moreover, we further validated the function of the lncRNA PRR34-AS1/miR-498/MIEF2 axis in mice. Compared with that in the shRNA-PRR34-AS1 group, the tumor volume was significantly reduced in the sh-PRR34-AS1+miR-498 antagomir and sh-PRR34-AS1+ov-MIEF2 groups ( *P*<0.01; Figure 7A,B). The mRNA expression levels of PRR34-AS1 and MIEF2 in the shRNA-PRR34-AS1 group were notably lower, while the expression level of miR-498 was markedly greater than that in the shRNA-Control group ( *P*<0.05). Compared with that in the shRNA-PRR34-AS1 group, the mRNA level of miR-498 in the sh-PRR34-AS1+miR-498 antagomir group was significantly decreased, but the mRNA level of MIEF2 was significantly increased ( *P* <0.05; Figure 7C).

Immunohistochemistry was used to detect the expression levels of glycolytic pathway-related kinases ( Figure 7D,E). Compared with those in the shRNA-PRR34-AS1 group, the expressions of GLUT1, HK2, PKM2, and LDHA were upregulated in the sh-PRR34-AS1+miR-498 antagomir and sh-PRR34-AS1+ov-MIEF2 groups. TEM revealed a significant reduction in the number of mitochondria, irregularly shaped nuclei, and uneven chromatin distribution in tumor cells in the sh-PRR34-AS1 group, which were greater in the sh-PRR34-AS1+miR-498 antagomir and sh-PRR34-AS1-ov-MIEF2 groups ( Figure 7F). The protein expression levels of Fis1, Drp1, and MIEF2 in the shRNA-PRR34-AS1 group were the lowest and were notably lower than those in the shRNA-Control group ( *P*<0.01). Compared to those in the shRNA-PRR34-AS1 group, the expression levels of these proteins were greater in the sh-PRR34-AS1+miR-498 antagomir group and sh-PRR34-AS1+ov-MIEF2 group ( *P*<0.01; Figure 7G). Overall, the lncRNA PRR34-AS1 affects mitochondrial kinetics and glycolytic reprogramming in mice with HCC through the targeted regulation of MIEF2 by miR-498.

## Discussion

Liver hepatocellular carcinoma (LIHC) is a malignant tumor with a very high mortality rate [17]. The prevention and treatment of liver cancer are still limited by the lack of effective treatments. To prevent, diagnose, and treat liver cancer effectively, it is vital to strengthen research on its pathogenesis and identify new targets for treatment. In our study, high PRR34-AS1 expression in HCC patients was verified, and patients with high PRR34-AS1 expression had shorter survival than those with low PRR34-AS1 expression. Furthermore, in HCC cells with high PRR34-AS1 expression, the proliferative activity, migratory ability and invasive capacity of the cells were significantly enhanced, revealing that PRR34-AS1 accelerates the development of HCC. Finally, we found that PRR34-AS1 can sponge miR-498 and that MIEF2 is a target of miR-498. PRR34-AS1 may regulate MIEF2 expression by sponging miR-498, mediating mitochondrial division, promoting glycolytic reprogramming in HCC cells, and ultimately promoting HCC progression.

Long noncoding RNAs (lncRNAs) exert their gene transcriptional regulatory functions through epigenetic regulatory mechanisms [18]. Various physiological and pathological processes are regulated by lncRNAs, and abnormal expression of lncRNAs is often observed in cancer [ 19, 20]. LncRNAs can regulate cancer cell proliferation, migration, invasion and other malignant behaviors, as well as epithelial–mesenchymal transition [ 21, 22]. High level of the lncRNA PRR34-AS1 is expressed in HCC, and the adsorption of microRNA-498 can upregulate the expression of FOXO3 and accelerate the development of HCC [5]. PRR34-AS1 induces miR-498 sponging and promotes TOMM20-ITGA6-mediated HCC progression. Taken together, our findings indicate that PRR34-AS1 is a major factor in the development and occurrence of HCC.

A significant difference exists between tumor cells and normal cells in terms of their metabolism. Even in the presence of sufficient oxygen, tumor cells still exhibit abnormally active glucose uptake and glycolytic activity and simultaneously produce large amounts of lactate and ATP [23]. In our study, glucose uptake and the lactic acid and ATP contents of liver cancer cells were significantly increased, and glycolytic pathway-related kinase protein expression was increased when PRR34-AS1 expression was elevated. That is, the level of glycolysis increased. Thus, PRR34-AS1 promotes glycolytic reprogramming in HCC cells.

Mitochondria are involved in the regulation of various signaling pathways and energy metabolism homeostasis in cells through mitochondrial quality control (MQC). Glycolytic metabolic reprogramming of cells occurs as a result of mitochondrial function being regulated by MQC homeostasis, among which mitophagy and mitochondrial dynamics are major regulatory mechanisms [24]. The dynamic process of mitochondrial division and fusion is known as mitochondrial dynamics. Abnormal mitochondrial fusion and division are present in many malignant tumors and are characterized by significantly enhanced mitochondrial division, resulting in abnormal expression of key proteins regulating mitochondrial fusion and division, thus promoting cell proliferation [25]. Therefore, based on changes in mitochondrial dynamics, this study explored the mechanism by which PRR34-AS1 promotes glucose metabolism reprogramming in HCC cells. The dynamic-like protein Drp1, located in the cytosol, and the mitochondrial division protein Fis1, located in the outer membrane, regulate mitochondrial division. Previous studies have shown that Drp1 expression promotes the progression of malignant tumors, including lung cancer, liver cancer and pancreatic cancer [ 26, 27]. Upregulation of Drp1 expression can promote tumor growth and metastasis by activating glycolysis in pancreatic cancer cells [28]. Recent studies have demonstrated that mitochondrial division mediated by the mitochondrial dynamic-related protein Drp1 is a regulatory factor for enhanced glycolysis in HCC cells [25]. Consistent with the results of our study, mitochondrial division was increased in cells with high PRR34-AS1 expression. In cells with high PRR34-AS1 expression, mitochondrial division-related proteins were expressed at higher levels. Silencing of *PRR34-AS1* in mice decreased the expressions of mitochondrial division-associated proteins. The above results demonstrated that high PRR34-AS1 expression promotes mitochondrial division.

As an outer membrane protein of mitochondria, MIEF2 plays a role in the regulation of mitochondrial division [12]. However, it is still unclear how MIEF2 is expressed and biologically activated in human cancers. Researchers have shown that MIEF2 expression is notably increased in ovarian cancer and promotes cell growth and metastasis. This promoting effect may be due to mitochondrial disruption in ovarian cancer cells, which shifts the main glucose metabolism pathway from oxidative phosphorylation to glycolysis [29]. MIEF2 may play an important role in cancer development, mitochondrial division and glucose metabolism. We further detected the targeted binding of miR-498 to MIEF2 through a dual-luciferase reporter assay. The role of miRNAs in liver cancer physiopathology is well established [29]. Studies have confirmed that miR-934 and miR-498 are abnormally expressed in primary liver cancer [30]. Then, we explored the regulatory relationship of the lncRNA PRR34-AS1/miR-498/MIEF2 axis. HCC cells with low PRR34-AS1 expression exhibit suppressed proliferation, migration, invasion, glycolysis and mitochondrial division. However, inhibiting miR-498 or increasing the expression level of MIEF2 reversed this downwards trend, mediated mitochondrial division, promoted glucose metabolism reprogramming, and restored the proliferation and growth of liver cancer cells. This finding was further confirmed in mice. These results further demonstrated that the lncRNA PRR34-AS1/miR-498/MIEF2 axis can promote glucose metabolism reprogramming and drive the growth and invasion of LIHC cells by regulating mitochondrial division.

In summary, the lncRNA PRR34-AS1 regulates MIEF2 expression by sponging miR-498, which affects glucose metabolism and mitochondrial division, thus promoting the development of HCC. This study revealed the mechanism by which PRR34-AS1 promotes the growth and development of liver cancer cells. However, the relationship between PRR34-AS1 and the prognosis of HCC patients, as well as the potential of PRR34-AS1 as a tumor marker to predict the development of HCC, needs to be verified via the analysis of a large amount of clinical data.
